# OP21 Long-Term Cost-Effectiveness Of IDegLira Versus Basal-Bolus Insulin Therapy In Patients With Type 2 Diabetes In Chinese Setting

**DOI:** 10.1017/S0266462324000849

**Published:** 2025-01-07

**Authors:** Junling Weng, Dunming Xiao, Yingyao Chen

## Abstract

**Introduction:**

IDegLira is a fixed-ratio combination (FRC) comprising insulin degludec and the glucagon-like peptide-1 receptor agonist (GLP-1RA) liraglutide, used for treating uncontrolled type 2 diabetes mellitus (T2DM) patients. We aimed to compare the long-term clinical and cost outcomes between IDegLira and basal-bolus insulin therapy by analyzing data from the Chinese healthcare system.

**Methods:**

The IQVIA Core Diabetes Model was employed to compare the two treatments. Cohort characteristics were derived from DUAL II China in patients with uncontrolled T2DM on basal insulin. Efficacy data were sourced from a published indirect comparative analysis. Utilities, medication, and complications costs were taken from publications. All costs were calculated in Chinese yuan (CNY) and discounted to 2022. The long-term outcomes estimated by the model spanned 40 years and encompassed life years (LY), quality-adjusted life years (QALY), cumulative incidence of diabetes-related complications, time alive and free of complication, costs, incremental cost-effectiveness ratio (ICER), and a probabilistic sensitivity analysis.

**Results:**

IDegLira was associated with increased LY (12.46 vs 12.413), increased QALY (10.946 vs 10.669), and reduced costs (CNY408,580.94 [USD56,407.22] vs CNY586,937.81 [USD81,030.54]) compared to basal-bolus insulin therapy. IDegLira was dominant compared to basal-bolus insulin therapy and exhibited a reduction in the cumulative incidence of diabetes-related complications. Additionally, IDegLira extended the time alive and free of complications. The probabilistic sensitivity analysis showed with 100 percent accuracy that IDegLira is more cost effective as compared to basal-bolus insulin therapy.

**Conclusions:**

Based on this analysis, IDegLira can be fully regarded as a dominant treatment compared to basal-bolus insulin therapy in Chinese patients with uncontrolled T2DM on basal insulin.

